# Prediction of linear B-cell epitopes based on protein sequence features and BERT embeddings

**DOI:** 10.1038/s41598-024-53028-w

**Published:** 2024-01-30

**Authors:** Fang Liu, ChengCheng Yuan, Haoqiang Chen, Fei Yang

**Affiliations:** 1 School of Humanistic Medicine, Anhui Medical University, Hefei, 230032 Anhui China; 2 School of Biomedical Engineering, Anhui Medical University, Hefei, 230030 Anhui China

**Keywords:** Protein sequence analyses, Data processing

## Abstract

Linear B-cell epitopes (BCEs) play a key role in the development of peptide vaccines and immunodiagnostic reagents. Therefore, the accurate identification of linear BCEs is of great importance in the prevention of infectious diseases and the diagnosis of related diseases. The experimental methods used to identify BCEs are both expensive and time-consuming and they do not meet the demand for identification of large-scale protein sequence data. As a result, there is a need to develop an efficient and accurate computational method to rapidly identify linear BCE sequences. In this work, we developed the new linear BCE prediction method LBCE-BERT. This method is based on peptide chain sequence information and natural language model BERT embedding information, using an XGBoost classifier. The models were trained on three benchmark datasets. The model was training on three benchmark datasets for hyperparameter selection and was subsequently evaluated on several test datasets. The result indicate that our proposed method outperforms others in terms of AUROC and accuracy. The LBCE-BERT model is publicly available at: https://github.com/Lfang111/LBCE-BERT.

## Introduction

B cells, also known as B lymphocytes, play an extremely important role in the mammalian immune response. They differentiate into plasma cells in response to antigenic stimulation by bacteria and viruses, producing antibodies to combat bacterial and viral infections^1^. The fragment of the antigen molecule that specifically binds to the B-cell surface receptor or antibody is called the B-cell epitope (BCE)^2^. B-cell epitopes can either be consecutive amino acid residues in the antigen protein sequence, known as linear epitopes, or they can be discontinuous amino acid residues that interact with each other to fold the protein sequence into a three-dimensional conformational structure, known as conformational epitopes^3,4^.

The identification of BCE has greatly contributed to the development of biomedicine, for example, in the overall understanding of immune response mechanisms with respect to the design and development of relevant vaccines^5,6^. Experimental methods for identifying BCEs in the field of biology include X-ray crystallography, cryo-EM, nuclear magnetic resonance, hydrogen–deuterium exchange coupled to mass spectroscopy, and peptide-based approaches, etc.^7^ However, these methods are generally expensive, time-consuming, and labour-intensive^8^. The volume of biological data has grown rapidly in recent years and traditional experimental methods can no longer cope with such a large volume of data. Therefore, there is need to develop sequence-based computational methods to identify potential BCEs quickly and accurately^9^. Various computational methods have been published for predicting conformational or linear BCEs. Although 90% of these BCEs are for conformational epitopes and only 10% are for linear BCEs, linear BCEs have remained a focus of research in recent years^10,11^.

Early prediction methods for BCE, such as Antigenic^12^, PEOPLE^13^, BEPITOPE^14^, and BcePred^15^, only utilized the physicochemical properties of amino acids. With the development of computer technology, researchers have combined these methods with machine learning techniques to construct models that incorporate multiple physicochemical properties of proteins, resulting in new prediction methods. For example, the BepiPred^16^ predictor utilises a combined approach that incorporates the amino acid propensity scale and hidden Markov models (HMM). Chen et al.^17^ refined the single amino acid propensity scale by creating the Amino Acid Pairs (AAP) antigenicity scale. Experiments based on a support vector machine (SVM) classifier showed that the AAP antigenicity scale approach outperformed other methods in BCE prediction. As a result, a new BCE predictor, AAPPred, was developed^18^. Among the various machine learning models that have been developed to predict BCE, SVM has emerged as the dominant model. Many researchers have used this model to train BCE predictors based on different datasets that combine a variety of amino acid features, such as BCPred^19^, FBCPred^20^, COBEpro^21^, SVMTrip^22^, LBtope^23^, IgPred^24^, APCpred^25^, and EpitopVec^26^. BepiPred-2.0^4^ and iLBE^27^ are other machine learning models that have been used to predict BCE. Both models use random forest(RF) for epistasis classification, while iBCE-EL^9^ uses extraordinarily randomized tree(ERT) for model training. Sahu T K et al.^28^ focused on evaluating 18 coding methods based on SVM and RF. In addition to traditional machine learning models, deep learning models have also been applied to BCE classification. For example, the predictor ABCpred^29^, developed in 2006, used a recurrent neural network(RNN). DLBEpitope^30^ and EpiDope^31^ which used feedforward neural network(FNN) and deep neural networks (DNN) training, respectively.

This study proposes a new method, LBCE-BERT, for predicting linear BCEs. The method combines traditional amino acid residue features, sequence features, and the semantics of the BERT (Bidirectional Encoder Representation from Transformers) model used in natural language processing embedding to form an optimal feature set. The effectiveness of the method was validated on multiple datasets.

## Materials and method

**Figure 1:**
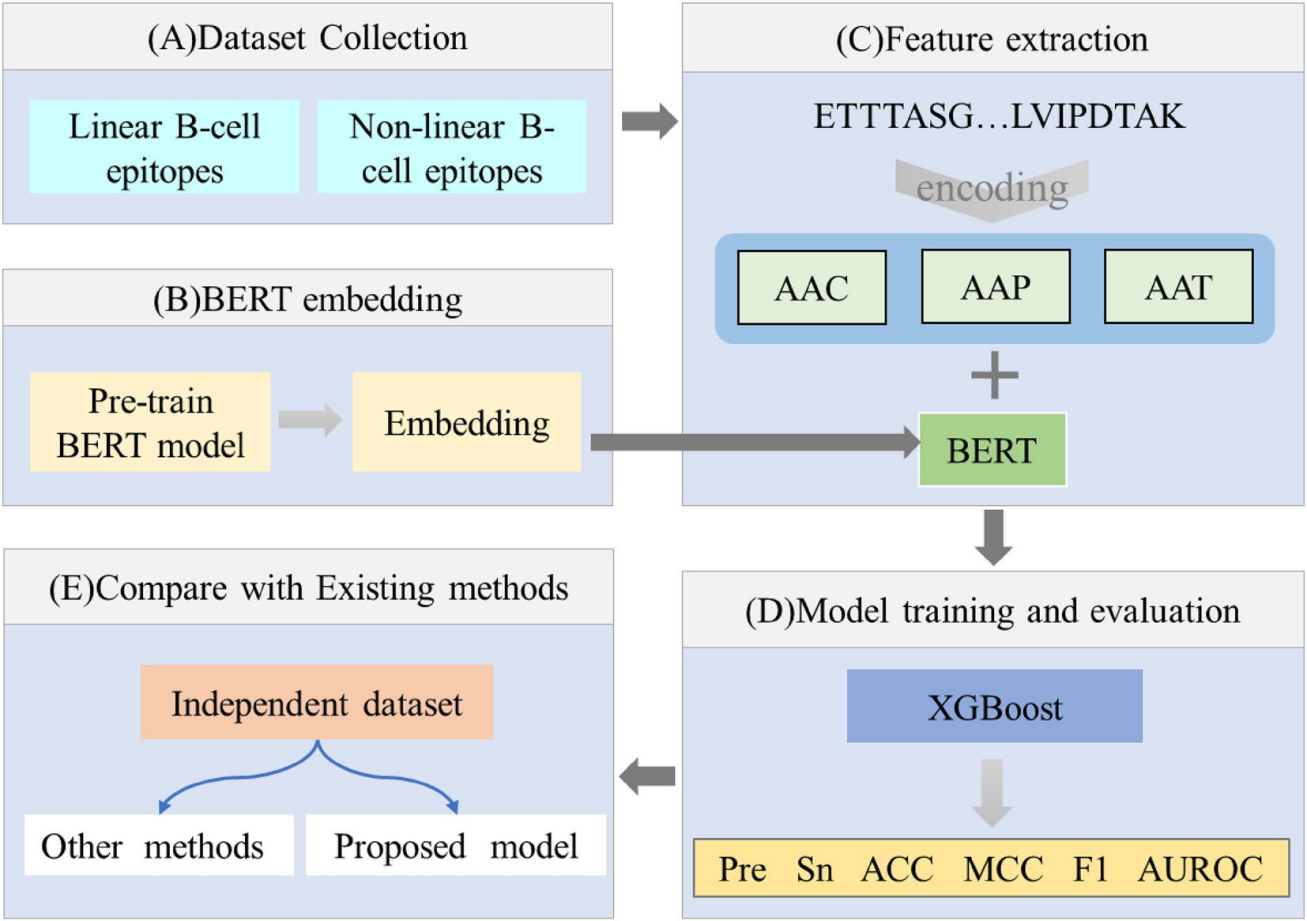
Overall flow of analysis in the present study. AAC: amino acid composition; AAP: amino acid pair scale; AAT: amino acid trimer scale; BERT: embeddings extracted from Bidirectional Encoder Representations from Transformers; Pre: precision; Sn: sensitivity; ACC: accuracy; MCC: Matthews correlation coefficient; F1: F1 score; and AUROC: area under the receiver operating characteristic (ROC) curve;

This study involved collecting datasets, extracting of protein sequence information and converting it into matrix information, and inputting the acquired matrix information into a machine learning model for training and hyperparameter optimization. Furthermore, the method was compared with existing approaches. The experimental structure of this work is shown in Fig. 1.

### Benchmark datasets

Most of the datasets used in the BCE predictors developed to date have been obtained from the Immune Epitope Database (IEDB)^32^ or the Bcipep^33^ database, where the data have been experimentally demonstrated. We collected the main benchmark datasets used by existing prediction models. Among them, the early proposed ABCPred^29^, Chen^17^ and BCPreds^19^ methods all collect data from the Bcipep database and construct their respective benchmark datasets. Other methods proposed subsequently, such as SVMTrip^34^, iBCE-EL^9^, BepiPred2.0^4^, DLBEpitope^30^ and EpiDope^31^, use data collected from IEDB. To ensure a fair and comprehensive comparison with previous methods, we used the benchmark datasets shown in Table 1. The dataset of ABCPred, Chen and BCPreds are all from the Bcipep database and the sequence length is fixed. Whereas the data of LBtope and iBCE-EL are all from the IEDB database and only the sequence length of LBtope is fixed. Therefore, we choose BCPreds, LBtope and iBCE-EL_training as the training dataset and the other datasets as the independent test set, respectively.

**Table 1:** The dataset used in the benchmarking of our method.

Dataset		Epitopes	Non-epitopes	Length	Homology cut-off value(%)	Source dataset
ABCPred		700	700	16	No	Bcipep
Chen		872	872	20	No	Bcipep
BCPreds		701	701	20	80	Bcipep
Blind387		187	200	15–75	No	Various
Lbtope		7824	7853	20	80	IEDB
iBCE-EL	training	4440	5485	5–25	70	IEDB
	independent	1110	1408	5–25	70	

#### BCPreds dataset

The BCPreds dataset was created by El-Manzalawy et al.^19^. First, sequence data for the B-cell epitopes were collected from the Bcipep database. Amino acid residues were then added or deleted at the ends of the original sequences so that each peptide chain was 20 amino acid residues in length. Next, 80% sequence identity was used as the threshold and duplicate or highly homologous sequences were removed using CD-HIT, leaving 701 epitope sequences as the final positive samples. The same number of non-epitope sequences of the same length were then obtained from Swiss-Prot^35^ as negative samples.

#### LBtope dataset

The LBtope dataset was one of the first datasets to obtain BCEs from the IEDB database, with five datasets created by Singh et al.^23^. In this work we have only used the ‘Lbtope_Fixed_non_redundant’ dataset, which uses the same data processing techniques as the BCPreds dataset.

#### iBCE-EL dataset

The iBCE-EL dataset was collected from the IEDB database. This dataset only includes sequence data that have been experimentally proven to be BCE epitopes twice or more. The original sequence length is maintained, and CD-HIT is used with a set threshold of 70% to reduce sequence homology. Unlike LBtope, the dataset does not add or subtract from the original sequence length.

### Feature representation of peptides

#### Amino acid composition

Amino acid composition (AAC)^26^ is a representation of the frequency of occurrence of each amino acid in a segment of a peptide chain, which can be expressed as

1 $$\begin{array}{c}{F}_{AAC}=\left({f}_{1},{f}_{2},{f}_{3},\dots ,{f}_{20}\right)\end{array}$$

where ${f}_{i}=\frac{{R}_{i}}{N}$ ($i=\mathrm{1,2},3,\dots ,20$) refers to the proportion of this amino acid in this peptide chain, ${R}_{i}$ refers to the $i$th amino acid and N refers to the length of this peptide chain.

#### Amino acid pair antigenicity scale

The Amino Acid Pair (AAP) antigenicity scale was first proposed and used by Chen et al.^17^. First, the ratio of the frequency of occurrence of amino acid pairs in positive samples to the frequency of occurrence of amino acid pairs in negative samples was calculated, and the resulting ratio was logarithmically normalised to [− 1, 1]. Positive samples were obtained from the Bcipep database and negative samples were obtained from the Swiss-Prot^36^ database, where negative samples were selected for all protein sequence information in the database except for B-cell epitope sequences. AAP antigenicity scale is calculated as

2 $$\begin{array}{c}{R}_{AAP}={\text{log}}\left(\frac{{f}_{AAP}^{+}}{{f}_{AAP}^{-}}\right)\end{array}$$

where ${f}_{AAP}^{+}$ and ${f}_{AAP}^{-}$ are the occurrences of specific dipeptides in BCEs and non-BCEs, respectively.

#### Amino acid trimer antigenicity scale

The Amino Acid Trimer (AAT) antigenicity scale was first used by researchers in SVMTrip^34^ and is similar to the AAP antigenicity scale, except that it targets amino acid trimers rather than amino acid pairs and results are also normalised to [− 1,1] on the propensity scale. The AAT antigenicity scale is calculated as

3 $$\begin{array}{c}{R}_{AAT}={\text{log}}\left(\frac{{f}_{AAT}^{+}}{{f}_{AAT}^{-}}\right)\end{array}$$

where ${f}_{AAT}^{+}$ and ${f}_{AAT}^{-}$ are the occurrences of specific tripeptide in BCEs and non-BCEs, respectively.

#### Sequence embeddings of BERT

The BERT (bidirectional encoder representations from transformers) model proposed by Devlin et al.^37^, has gained significant recognition in the field of natural language processing due to its exceptional performance. As a result, it has been widely adopted across various domains, including bioinformatics. For example, Qiao et al.^38^ developed BERT-Kcr, a predictor for identifying protein lysine crotonylation sites use the BERT model. Similary Liu et al.^39^ created BERT-Kgly, a novel predictor for lysine glycosylation sites by combining features extracted from a pre-trained protein language BERT model with a deep learning model.

BERT focuses on using a new masked language model (MLM) to train a bidirectional transformer for creating deep bidirectional language representations. The coding layer of this mechanism uses a multi-head self-attention approach to process both left and right contexts simultaneously, allowing for parallel processing of all words in a sentence. The attention mechanism is based on three main concepts: Query (the target word, or the annotated word to be generated), Value (the original Value representation of each word in the context), and Key (the Key vector representation of each word in the context). In the multi-headed self-attentive mechanism, it first uses $h$ linear transformations with different parameters to project Query, Value and Key, followed by inputting each of the transformed $h$ sets of vectors to the self-attentive layer. After the self-attentive layer, the model obtains different attentional results. These results are then combined to create an information representation of the different subspaces. The main calculation procedure is shown in the following equation:

4 $$\begin{array}{c}MultiHead\left({\text{Q}},{\text{K}},{\text{V}}\right) = Concat\left({{\text{head}}}_{1},\cdots ,{{\text{head}}}_{{\text{h}}}\right){{\text{W}}}^{{\text{O}}}\end{array}$$

5 $$\begin{array}{c}{{\text{head}}}_{i} = Attention\left({{\text{QW}}}_{{\text{i}}}^{{\text{Q}}},{{\text{KW}}}_{{\text{i}}}^{{\text{K}}},{{\text{VW}}}_{{\text{i}}}^{{\text{V}}}\right)\end{array}$$

6 $$\begin{array}{c}Attention\left({\text{Q}},{\text{K}},{\text{V}}\right) = softmax\left(\frac{{{\text{QK}}}^{{\text{T}}}}{\sqrt{{{\text{d}}}_{{\text{k}}}}}\right)V\end{array}$$

where Q, K, V represent the three vectors Query, Key, Value. ${d}_{k}$ is the dimension of Key, and ${W}_{i}^{Q}$, ${W}_{i}^{V}$, ${W}_{i}^{V}$ and ${W}^{O}$ are parameter matrices^40^. When a sentence is fed into the BERT model, the input vector for each word consists of three components: token embedding, segment embedding and position embedding. Where the token embedding represents each word present in the dictionary, encoded based on a different partitioning method. The fragment embedding indicates whether the word belongs to the first or second half of the sentence. The formula for encoding the positional embedding, which represents the position of a word in a sentence, is as follows:

7 $$\begin{array}{c}{{\text{PE}}}_{\left(pos,2i\right)} = sin\left(\frac{pos}{{10000}^{\frac{2i}{{d}_{model}}}}\right)\end{array}$$

8 $$\begin{array}{c}{{\text{PE}}}_{\left(pos, 2i+1\right)} = cos\left(\frac{pos}{{10000}^{\frac{2i}{{d}_{model}}}}\right)\end{array}$$

where *pos* is the position, $i$ is the component position of the vector *d*_*model*_ represents the dimension of the vector^37^. Then, context-dependent features can be obtained from various encoder layers of the model.

### Machine learning methods

The XGBoost (eXtreme Gradient Boosting)^41^ classifier was used in this study to build the model. It is based on a modification of the gradient boosting decision tree (GBDT)^42^. This classifier is a modified version of the gradient boosting decision tree (GBDT), which combines multiple regression trees to predict values that are as close to the true values as possible and have strong generalisation power. The model has two main advantages: it is regularised to prevent overfitting and supports parallelisation, which can greatly speed up training.

### Evaluation metrics

This study employed five commonly used evaluation metrics to assess the model: accuracy (ACC), precision (Pre), sensitivity (Sn), F1 score (F1) and Matthews correlation coefficient (MCC). Additionally, we also calculated the receiver operating characteristic (ROC) curve and calculated the area under the receiver operating characteristic (ROC) curve (AUROC).

9 $$\begin{array}{c}Sn=\frac{TP}{TP+FN}\end{array}$$

10 $$\begin{array}{c}Pre=\frac{TP}{TP+FP}\end{array}$$

11 $$\begin{array}{c}ACC=\frac{TP+TN}{TP+TN+FP+FN}\end{array}$$

12 $$\begin{array}{c}F1=\frac{2TP}{2TP+FP+FN}\end{array}$$

13 $$\begin{array}{c}MCC=\frac{TP\times TN-FP\times FN}{\sqrt{\left(TP+FP\right)\left(TP+FN\right)\left(TN+FP\right)\left(TN+FN\right)}}\end{array}$$

where TP, TN, FP and FN signify the numbers of true positives, true negatives, false positives and false negatives, respectively.

## Results

### Sequence discrepancy between positive and negative samples in the benchmark dataset

**Figure 2:**
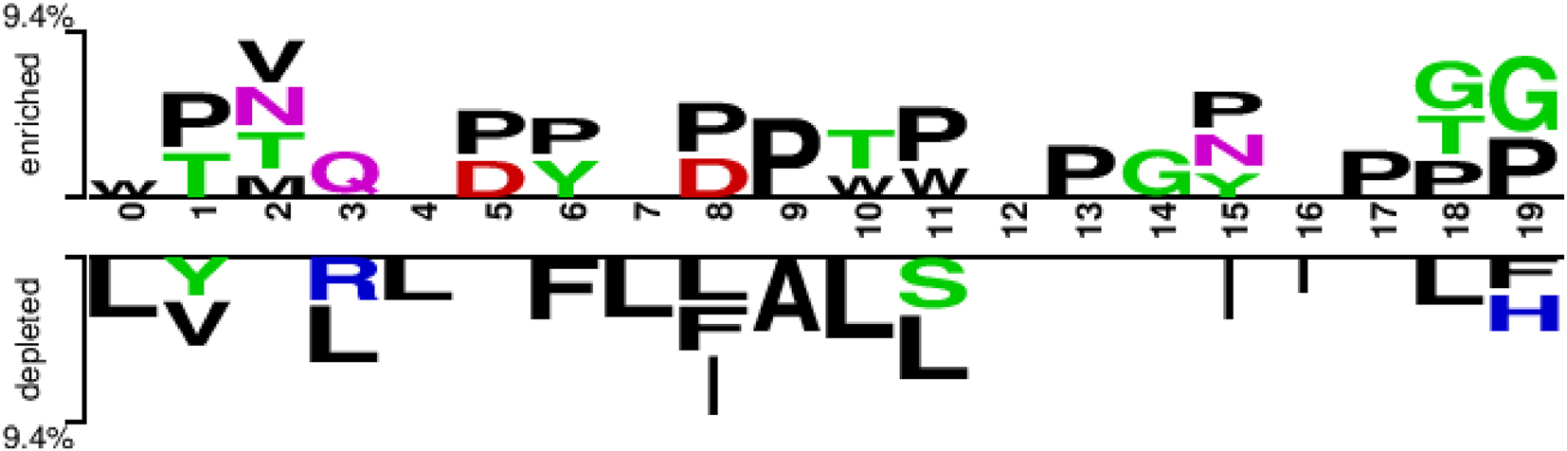
Overall sequence pattern discrepancy between positive and native samples illustrated by Two Sample Logo^43^ in the benchmark dataset BCPreds.

**Figure 3:**
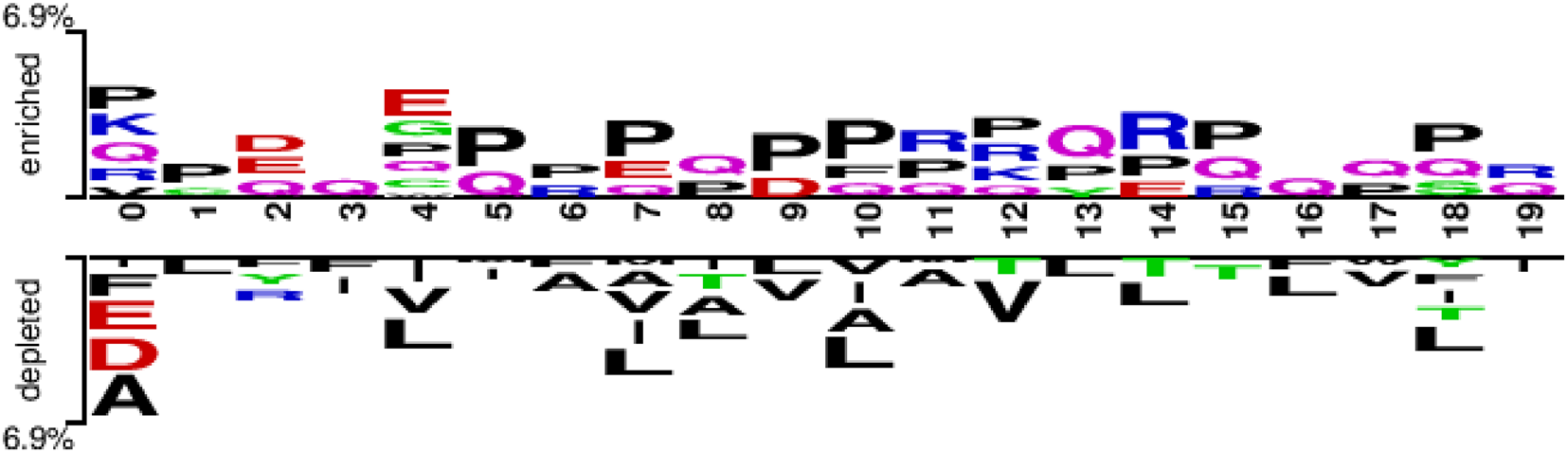
Overall sequence pattern discrepancy between positive and native samples illustrated by Two Sample Logo^43^ in the benchmark dataset LBtope.

We developed a machine learning method based on peptide sequence information to distinguish between BCEs and non-BCEs. The assumption of different sequence patterns for positive and negative samples was taken into account. For overall pattern differences, we visualized them by two sample markers, the distribution and preference of epitope and non-epitope residues^43^. Figure 2 illustrates that residue P is significantly enriched in several positions in the positive samples, while residues T, G, and D are enriched to varying degrees. In contrast, residue L is significantly depleted in several positions in the negative samples, along with residues I and F. The overall enrichment or depletion rate for specific sequential positions was 9.4%. Figure 3 shows an enrichment or depletion ratio of 6.9% for specific sequence positions in the benchmark dataset LBtope. Residues P and Q are enriched to a higher degree in positive samples, while residues L, V, and A are depleted to a higher degree in negative samples. It is worth noting that the sum of the enrichment and depletion rates of the different residues at each position implies a difference between the positive and negative samples at that position.

### Model based on embeddings of pretrained BERT models

In this work, we directly used the BERT pre-training model constructed by Zhang et al.^44^ based on 556,603 protein sequences to encode peptide chain sequences and extract their embeddings of tokens as features. To experiment the effectiveness of this feature encoding approach, we extracted the embedding of the marker ‘CLS’ and the average embedding of all amino acids in the entire peptide chain as features from the epitope and non-epitope sequences in the BCPreds, iBCE-EL_training and LBtope datasets, respectively. The ‘CLS’ is always the first token of each classification sequence, as described in Devlin et al.^45^. Next, we built a model to predict linear BCE using the XGBoost classifier and trained it with various features. Table 2 shows the results of the five-fold cross-validation of the different models. The results show that the model trained with the embedding of the token ‘CLS’ consistently outperforms the model trained with the average embedding of all amino acids in the entire peptide chain, regardless of which dataset the model is trained on in BCPreds, iBCE-EL_training and LBtope. Therefore, in the next work, we will only use the embedding of the token ‘CLS’ from the protein BERT pre-training model as features for our experiments. The experimental results based on the BCPreds dataset were optimal in the above three datasets, and the combination of using embedding of the token ‘CLS’ as a feature to train the model achieved an AUC value of 0.693 and an ACC value of 0.629 for the five-fold cross-validation. Using the average embedding of all amino acids as a feature, the model achieved a five-fold cross-validation AUC value of 0.671 and an ACC value of 0.619.

**Table 2:** Cross-validation results based on two kinds of BERT embedding.

	Token^a^	Pre	Sn	F1	ACC	MCC	AUROC
BCPreds	‘CLS’	0.634	0.611	0.622	0.629	0.259	0.693
	‘avg’	0.624	0.601	0.612	0.619	0.238	0.671
iBCE-EL_training	‘CLS’	0.593	0.450	0.511	0.615	0.210	0.643
	‘avg’	0.587	0.440	0.503	0.610	0.199	0.638
LBtope	‘CLS’	0.566	0.540	0.552	0.565	0.128	0.589
	‘avg’	0.548	0.530	0.538	0.548	0.094	0.571

^a^‘avg’ means the average of the embeddings of residues in the epitope.

### Evaluate model performance based on different datasets

The AAC, AAP, AAT and embedding are combined with the token ‘CLS’ in the protein BERT pre-training model. This is then input into XGBoost for training to find hyperparameters. It is important to note that although variable-length linear BCEs exist in these datasets, these four feature representations enable the extraction of variable-length linear BCEs as fixed-dimension matrix information. As a result, the variable-length linear BCE does not affect the training model. We trained three different BCE prediction models using BCPreds, LBtope, and iBCE-EL_training datasets. These models are labelled LBCE-BERT(BCPreds), LBCE-BERT(LBtope) and LBCE-BERT(iECB-EL). To evaluate the model’s performance, we performed a five-fold cross-validation and independent dataset validation based on seven different datasets (BCPreds dataset, Chen dataset, ABCPred dataset, Blind387 dataset, LBtope dataset, iBCE-EL_training dataset, iBCE-EL_independent dataset), where the evaluation metrics for models are calculated as shown in section “Evaluation metrics”. The following are the results and correlation analysis of the five-fold cross-validation of the three models.

To observe the test performance of model strained on BCPreds, LBtope, and iBCE-EL_training on other datasets. We plotted AUROC curves for BCPreds, Chen, ABCPred, Blind387, LBtope, iBCE-EL_training, and iBCE-EL_independent for their performance on the three models are shown in Figs. S1–S7, respectively.

#### Evaluation of model LBCE-BERT(BCPreds)

Table 3 shows that our proposed method, based on the BCPreds dataset with five-fold cross-validation, has an AUROC of 0.924. This is a 3% improvement over the EpitopVec model, which was also trained on the same dataset. We then tested this model on other datasets and found that it performed well in both Chen and ABCPred. As shown in Table 4, our method achieves a higher AUROC of 0.990 on the Chen dataset compared to EpitopVec (the current optimal method) which achieved 0.959. Additionally, our method has an ACC of 0.941, representing a 21.6% improvement over the original method. On the Chen dataset, the models’ prediction accuracy ranged from 0.494 to 0.941, the lowest performing methods were iBCE-EL and LBtope, with ACC values of 0.494 and 0.533, respectively. In the ABCPred dataset (Table 5), the accuracy of the original method (ABCPred) is only 0.6593, which is lower than that of AAP(0.7314), BCPreds(0.7457), and EpitopVec(0.856). Additionally, LBCE-BERT(BCPreds) has the highest AUROC value of 0.934 among all methods, indicating that our model’s performance is stable and efficient. The Blind387 dataset was also tested, and the results are shown in Table 6. This dataset consist of epitopes derived from viruses and was obtained from published literature. Our model’s performance on this dataset was mediocrely, with an ACC of 0.705, which is 4.1% higher than the original method used to build the dataset but slightly lower than the optimal method, EpitopVec. Similarly, the AUROC value of 0.757 is only 2.2% lower than the optimal method. Chen, ABCPred, LBtope and iBCE-EL are some of the earlier methods for obtaining BCE datasets using the IEDB database, compared to BCPreds. According to the results in Table 7, LBCE-BERT(BCPreds), BCPreds, iBCE-EL, and EpitopVec(BCPreds) have similar accuracy on the LBtope dataset. However, on the training and independent test sets of iBCE-EL (Table 8), EpitopVec(BCPreds) slightly outperformed LBCE-BERT(BCPreds).

**Table 3:** Multi-method prediction results on the BCPreds dataset.

Method^a^	Pre^b^	Sn^b^	F1^b^	MCC^b^	ACC^b^	AUROC^b^
ABCPred						0.643
AAP					0.6405	0.7
BCPreds		0.73		0.360	0.679	0.758
LBtope					0.5157	
iBCE-EL	0.49	**0.97**	0.33	− 0.009	0.4871	0.576
EpitopeVec (BCPreds)	0.808	0.807	0.807	0.618	0.808	0.894
EpitopeVec (LBtope)	0.559	0.692	0.619	0.151	0.573	0.645
EpitopeVec (iECB-EL)	0.574	0.572	0.573	0.148	0.574	0.602
**LBCE-BERT** **(BCPreds)**	**0.872**	0.820	**0.844**	**0.701**	**0.850**	**0.924**
**LBCE-BERT** **(LBtope)**	0.664	0.482	0.559	0.248	0.619	0.641
**LBCE-BERT** **(iECB-EL)**	0.619	0.534	0.573	0.207	0.603	0.643

^a^To facilitate understanding, the highest value in each dataset is shown in bold. The methods shown in bold are those proposed in this work.

^b^Blank cells indicate that the indicator scores reported in the original publication do not exist.

**Table 4:** Multi-method prediction results on the Chen dataset.

Method^a^	Pre^b^	Sn^b^	F1^b^	MCC^b^	ACC^b^	AUROC^b^
AAP		0.61		0.366	0.7109	0.7
AAP + scales		0.64		0.404	0.7254	
LBtope					0.5333	
iBCE-EL	0.5	0.96	0.35	− 0.036	0.494	0.528
EpitopeVec (BCPreds)	0.849	0.932	0.889	0.770	0.883	0.959
EpitopeVec (LBtope)	0.563	0.714	0.630	0.167	0.580	0.658
EpitopeVec (iECB-EL)	0.573	0.567	0.570	0.145	0.572	0.591
**LBCE-BERT** **(BCPreds)**	**0.911**	**0.977**	**0.943**	**0.884**	**0.941**	**0.990**
**LBCE-BERT** **(LBtope)**	0.650	0.460	0.539	0.222	0.606	0.639
**LBCE-BERT** **(iECB-EL)**	0.598	0.525	0.559	0.173	0.586	0.616

^a^To facilitate understanding, the highest value in each dataset is shown in bold. The methods shown in bold are those proposed in this work.

^b^Blank cells indicate that the indicator scores reported in the original publication do not exist.

**Table 5:** Multi-method prediction results on the ABCPred dataset.

Method^a^	Pre^b^	Sn^b^	F1^b^	MCC^b^	ACC^b^	AUROC^b^
ABCPred		0.67		0.466	0.6593	
AAP		0.50		0.518	0.7314	0.782
BCPreds		0.70		0.493	0.7457	0.801
LBtope					0.5790	
iBCE-EL	0.51	**0.96**	0.42	0.112	0.527	0.588
EpitopeVec (BCPreds)	0.836	0.884	0.860	0.713	0.856	0.928
EpitopeVec (LBtope)	0.612	0.766	0.680	0.289	0.640	0.719
EpitopeVec (iECB-EL)	0.686	0.353	0.466	0.219	0.596	0.615
**LBCE-BERT** **(BCPreds)**	**0.873**	0.886	**0.879**	**0.757**	**0.879**	**0.934**
**LBCE-BERT** **(LBtope)**	0.637	0.454	0.530	0.204	0.598	0.617
**LBCE-BERT** **(iECB-EL)**	0.619	0.520	0.565	0.203	0.600	0.625

^a^To facilitate understanding, the highest value in each dataset is shown in bold. The methods shown in bold are those proposed in this work.

^b^Blank cells indicate that the indicator scores reported in the original publication do not exist.

**Table 6:** Multi-method prediction results on the Blind387 dataset.

Method^a^	Pre^b^	Sn^b^	F1^b^	MCC^b^	ACC^b^	AUROC^b^
ABCPred		0.72			0.6641	
AAP		0.64		0.292	0.6460	0.689
BCPreds		0.66		0.318	0.6589	0.699
iBCE-EL	0.44	**0.84**	0.32	− 0.227	0.434	0.501
EpitopeVec (BCPreds)	**0.759**	0.588	**0.663**	**0.427**	**0.711**	**0.779**
EpitopeVec (LBtope)	0.591	0.834	0.692	0.316	0.641	0.755
EpitopeVec (iECB-EL)	0.733	0.572	0.643	0.389	0.693	0.726
**LBCE-BERT** **(BCPreds)**	**0.759**	0.572	0.652	0.418	0.705	0.757
**LBCE-BERT** **(LBtope)**	0.618	0.519	0.564	0.223	0.612	0.656
**LBCE-BERT** **(iECB-EL)**	0.583	0.524	0.552	0.175	0.589	0.626

^a^To facilitate understanding, the highest value in each dataset is shown in bold.

^b^Blank cells indicate that the indicator scores reported in the original publication do not exist.

**Table 7:** Multi-method prediction results on the LBtope dataset.

Method^a^	Pre^b^	Sn^b^	F1^b^	MCC^b^	ACC^b^	AUROC^b^
BCPreds					0.5256	
LBtope					0.6486	0.69
iBCE-EL	0.51	**0.99**	0.39	0.135	0.522	0.619
EpitopeVec (BCPreds)	0.546	0.286	0.376	0.056	0.525	0.538
EpitopeVec (LBtope)	**0.684**	0.705	**0.694**	**0.381**	**0.690**	**0.755**
EpitopeVec (iECB-EL)	0.567	0.613	0.589	0.147	0.573	0.603
**LBCE-BERT** **(BCPreds)**	0.555	0.208	0.302	0.054	0.522	0.547
**LBCE-BERT** **(LBtope)**	0.665	0.686	0.675	0.342	0.671	0.733
**LBCE-BERT** **(iECB-EL)**	0.589	0.624	0.606	0.190	0.595	0.633

^a^To facilitate understanding, the highest value in each dataset is shown in bold. The methods shown in bold are those proposed in this work.

^b^Blank cells indicate that the indicator scores reported in the original publication do not exist.

**Table 8:** Multi-method prediction results on the iECB-EL dataset.

	Method^a^	Pre^b^	Sn^b^	F1^b^	MCC^b^	ACC^b^	AUROC^b^
iECB-EL training dataset	iBCE-EL		0.716		0.454	0.729	0.782
	EpitopeVec (BCPreds)	0.512	0.263	0.347	0.071	0.558	0.551
	EpitopeVec (LBtope)	0.551	**0.746**	0.634	0.259	0.615	0.715
	EpitopeVec (iECB-EL)	0.698	0.644	0.669	0.422	0.715	0.793
	**LBCE-BERT** **(BCPreds)**	0.507	0.240	0.325	0.062	0.556	0.540
	**LBCE-BERT** **(LBtope)**	0.586	0.563	0.575	0.243	0.627	0.667
	**LBCE-BERT** **(iECB-EL)**	**0.716**	0.710	**0.713**	**0.482**	**0.744**	**0.820**
iECB-EL independent dataset	iBCE-EL	0.66	**0.79**	**0.73**	0.454	0.734	0.786
	EpitopeVec (BCPreds)	0.519	0.250	0.338	0.082	0.567	0.566
	EpitopeVec (LBtope)	0.558	0.769	0.647	0.294	0.630	0.742
	EpitopeVec (iECB-EL)	0.682	0.647	0.664	0.412	0.711	0.785
	**LBCE-BERT** **(BCPreds)**	0.514	0.234	0.322	0.074	0.565	0.545
	**LBCE-BERT** **(LBtope)**	0.599	0.595	0.597	0.282	0.646	0.706
	**LBCE-BERT** **(iECB-EL)**	**0.722**	0.731	0.726	**0.508**	**0.757**	**0.828**

^a^To facilitate understanding, the highest value in each dataset is shown in bold. The methods shown in bold are those proposed in this work.

^b^Blank cells indicate that the indicator scores reported in the original publication do not exist.

#### Evaluation of model LBCE-BERT(LBtope)

Table 7 shows the results of the five-fold cross-validation based on the LBtope dataset. The model trained on this dataset performs slightly worse. On the BCPreds dataset (Table 3), the AUROC values for LBCE-BERT(LBtope), EpitopVec(LBtope), and ABCPred were 0.641, 0.645, and 0.643, respectively. The ACC values for LBtope and iBCE-EL were 0.5157 and 0.4871, respectively. In the Chen dataset (Table 4), our model is the second-best performing method, following the original proposed method. The lowest performing method is iBCE-EL, with an accuracy of only 0.494. LBCE-BERT(LBtope) did not perform well in both ABCPreds and Blind387, with AUROC values of 0.617 and 0.656 on these two datasets, respectively. Additionally, based on the LBtope dataset, LBCE-BERT (LBtope) achieved an AUROC of 0.733 and an ACC of 0.671. Other methods achieved ACC values ranging from 52.2 to 69%. Furthermore, as shown in Fig. 3, our model’s accuracy was slightly higher than EpitopVec(LBtope) but lower than the original method on both the training and independent test sets of iBCE-EL. However, our model’s AUROC was the lowest among the three models.

#### Evaluation of model LBCE-BERT(iBCE-EL)

In the five-fold cross-validation of our model based on the iBCE-EL training set, Table 8 shows that the AUROC value of 0.820 is 2.7% higher than the current optimal model EpitopeVec of 0.793. The original method presenting this data is only 0.782, proving that our method LBCE-BERT(iBCE-EL) is the current optimal model. In the iBCE-EL independent test set, the original method (iBCE-EL) achieved an AUROC of 0.786. EpitopeVec(iBCE-EL) achieved an AUROC of 0.785, while LBCE-BERT(iBCE-EL) achieved an AUROC of 0.828 and an ACC of 0.757. These results suggest that LBCE-BERT(iBCE-EL) has better generalisation ability. This model was also evaluated on other datasets. The results of LBCE-BERT (iBCE-EL) on each dataset BCPreds (Table 3), Chen (Table 4), ABCPred (Table 5), Blind387 (Table 6) and LBtope (Table 7), showed little various, with only a 0.017 different in ACC values and a 0.027 different in AUROC values. LBCE-BERT (iBCE-EL) achieved an ACC of 0.603 in the BCPreds dataset, 0.586 in Chen and 0.586 in ABCPred. In Blind387, LBCE-BERT (iBCE-EL) achieved an ACC value of 0.589, which is only better than the iBCE-EL method’s ACC of 0.434. Other methods, such as ABCPred(0.6641), AAP(0.6460), BCPreds(0.6589) and EpitopeVec(0.693), outperformed LBCE-BERT(iBCE-EL). On the LBtope dataset, our model achieved an ACC of 0.595, which is higher than BCPreds(0.5256), iBCE-EL(0.522) and EpitopeVec(0.573).

## Discussion and conclusions

In this study, we developed the LBCE-BERT method, a machine learning approach for predicting linear BCEs based on amino acid sequence features and the protein language model BERT embedding, and achieved good performance. The accurate prediction of linear BCEs using this method provides valuable insights for applications in biotechnology, such as the treatment and prevention of infectious diseases. We trained three different models based on the BCPreds, LBtope and iBCE-EL datasets respectively, and compared various methods, including the original method used to propose these datasets and the current optimal method for predicting BCE. The experimental results showed that the LBCE-BERT (BCPreds) and LBCE-BERT (iBCE-EL) models performed better than other models. However, the LBCE-BERT (LBtope) model performed slightly worse than EpitopVec. The difficult to classify sequences in the LBtope data set can be seen in the sequence analysis, showing lower overall enriched or depleted ratios for specific sequential positions compared to the BCPreds dataset. In contrast, the LBCE-BERT (iBCE-EL) achieves better performance on the dataset iBCE-EL, which is obtained from the same IEDB database as the dataset LBtope. This may be due to the original sequence being altered during addition or deletion processing, which can result in the loss of sequence information regarding the table position.

Furthermore, our model exhibited exceptional performance when validated with other datasets, indicating its strong generalization capability. However, during cross-validation on several datasets, all of which were derived from the Bcipep database, we observed a significant decrease in the predictive performance of one class of models. These models were primarily trained on datasets derived from the IEDB database. Similarly, when cross-validating on multiple datasets obtained from the IEDB database, we obtained the same results for models trained on datasets derived from the Bcipep database. These findings indicate that models trained on datasets from the same database may exhibit similarities. Therefore, it is important to diversify training sets for computational methods.

In conclusion, this study presents the LBCE-BERT method, which effectively predicts linear BCEs, and demonstrates its robust performance across multiple datasets. These findings contribute to the field of BCE prediction and offer valuable insights for future research in this area.

### Supplementary Information

Supplementary Information.

## Data Availability

All datasets and source codes of LBCE-XGB are publicly available on https://github.com/Lfang111/LBCE-BERT.
