# Supplementary material for: Prediction of linear B-cell epitopes based on protein sequence features and BERT embeddings
Source: Sci Rep. 2024 Jan 30;14:2464. doi: 10.1038/s41598-024-53028-w (PMC10828400; doi:10.1038/s41598-024-53028-w)
Supplement: Supplementary file 1 — Supplementary Information. [file 41598_2024_53028_MOESM1_ESM.pdf]

Supplementary Materials for

Prediction of linear B-cell epitopes based on protein sequence features and BERT embeddings

Fang Liu[1], ChengCheng Yuan[2], Haoqiang Chen[1] and Fei Yang[2]

[1]School of Humanistic Medicine, Anhui Medical University, Hefei, Anhui 230032, China

[2]School of Biomedical Engineering, Anhui Medical University, Hefei, Anhui 230030, China,

Contact: 438645814@qq.com

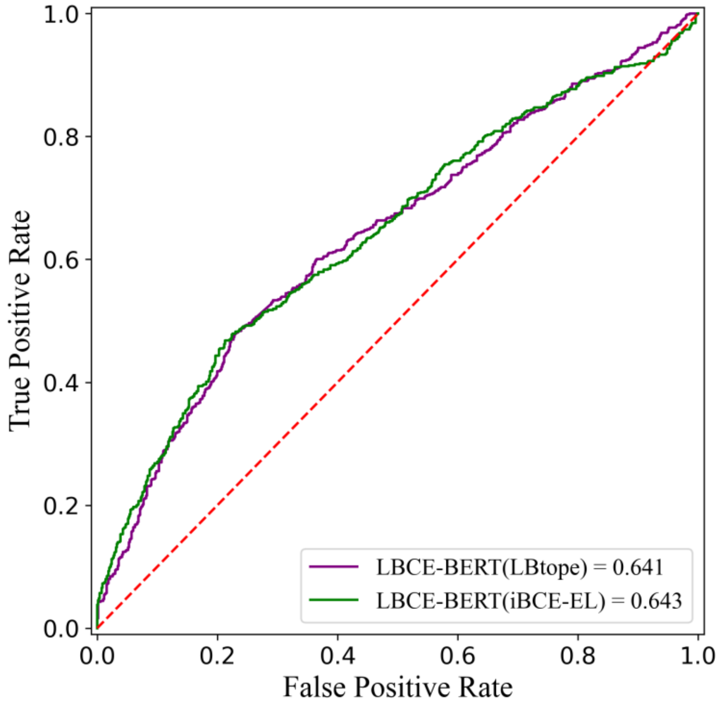

Figure S1. Results on the BCPreds dataset with AUROC curves. The model trained based on BCPreds is the result of five-fold cross-validation so the AUROC curve is not plotted.

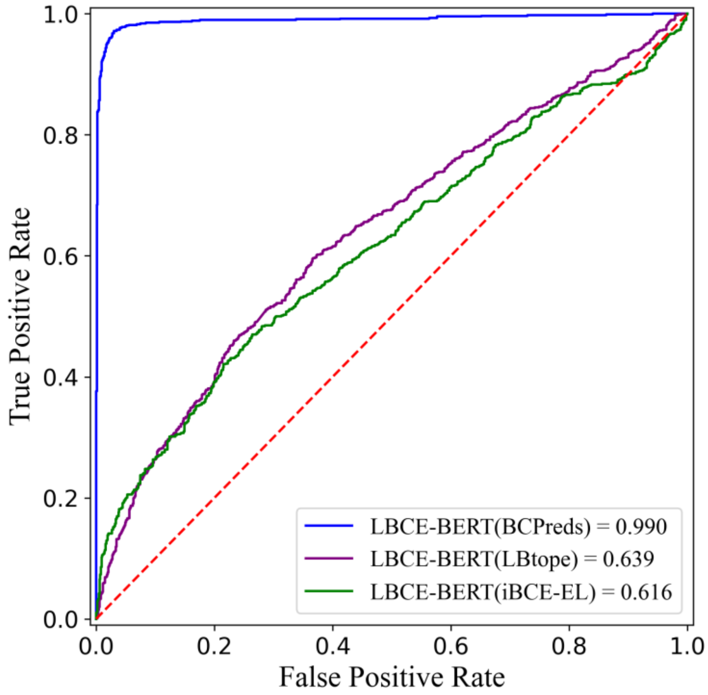

Figure S2. Results on the Chen dataset with AUROC curves.

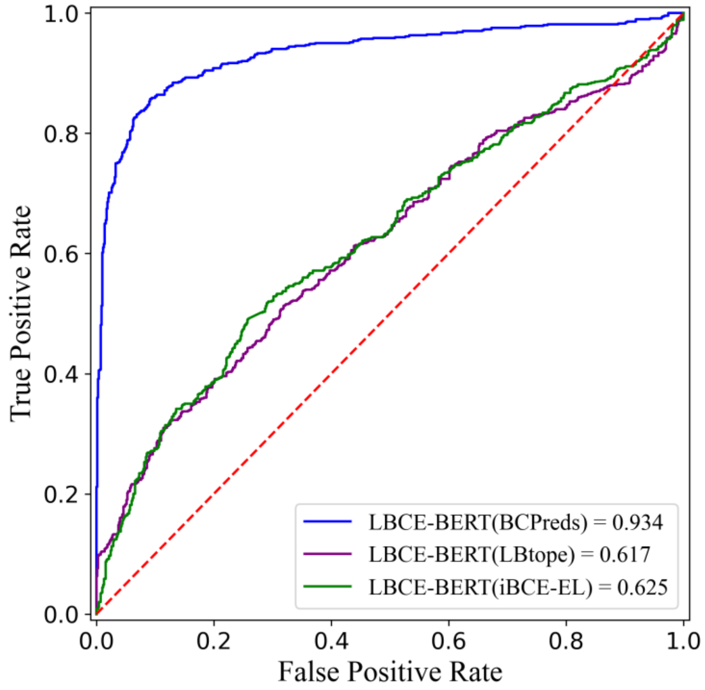

Figure S3. Results on the Chen dataset with AUROC curves.

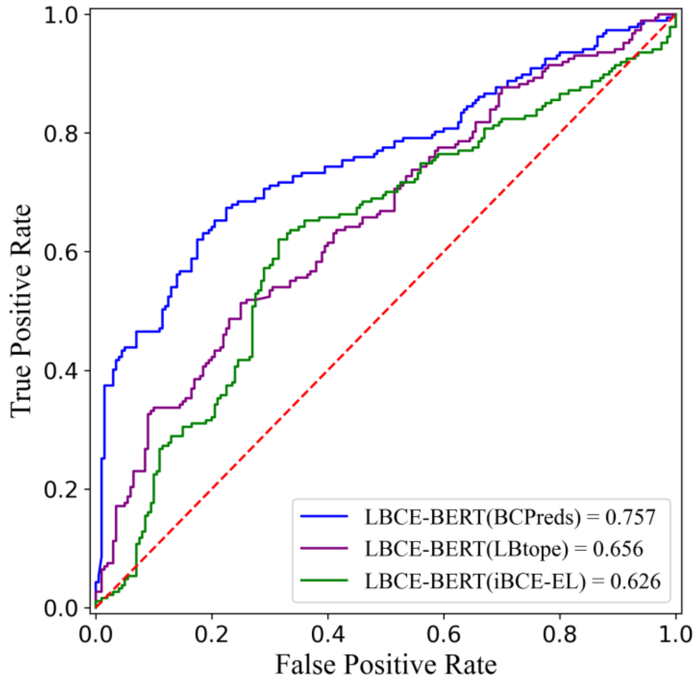

Figure S4. Results on the Blind387 dataset with AUROC curves.

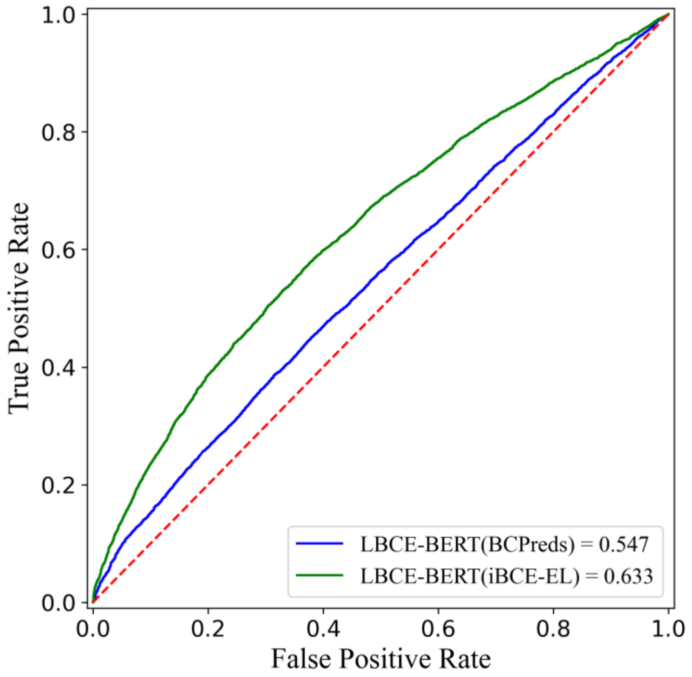

Figure S5. Results on the LBtope dataset with AUROC curves. The model trained based on LBtope is the result of five-fold cross-validation so the AUROC curve is not plotted.

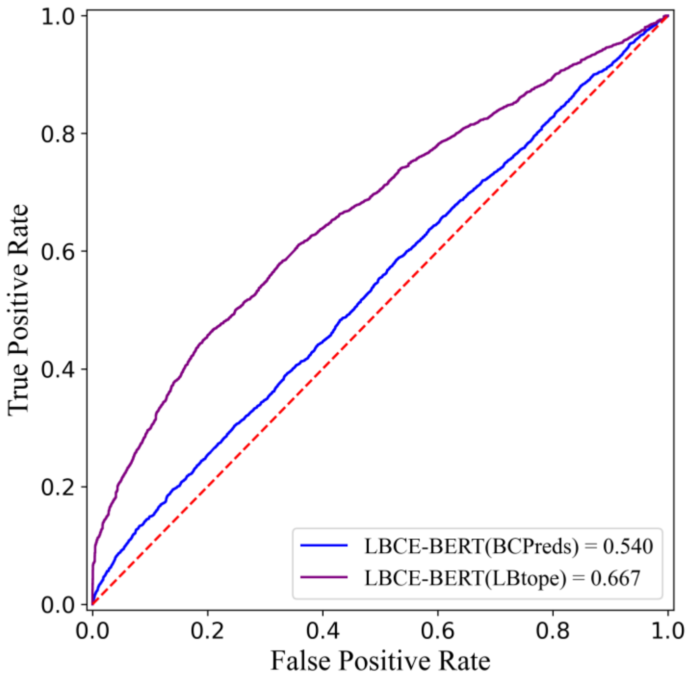

Figure S6. Results on the iBCE-EL_training dataset with AUROC curves. The model trained based on iBCE-EL_training is the result of five-fold cross-validation so the AUROC curve is not plotted.

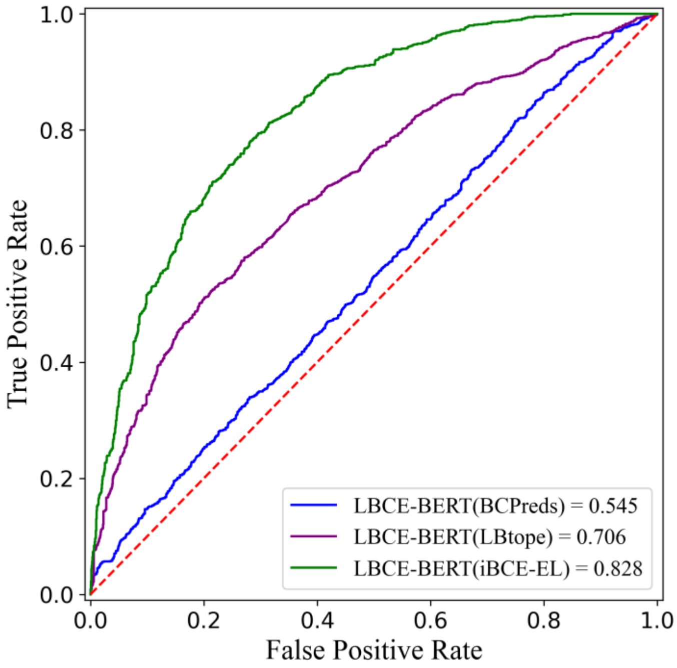

Figure S7. Results on the iBCE-EL_ independent dataset with AUROC curves.
